# Suspected illegal abortion and unsafe abortion leading to uterine rupture and incomplete abortion: A case report

**DOI:** 10.1016/j.amsu.2022.104933

**Published:** 2022-11-17

**Authors:** Ayush Anand, Ashwini Gupta, Punita Yadav, Pappu Rijal

**Affiliations:** a BP Koirala Institute of Health Sciences, Dharan, Nepal; b Department of Obstetrics and Gynaecology, BP Koirala Institute of Health Sciences, Dharan, Nepal

**Keywords:** Case report, Unsafe abortion, Illegal abortion, Uterine rupture, Incomplete abortion

## Abstract

**Introduction:**

Unsafe abortions are more prevalent in developing countries and countries with restrictive abortion laws, and can lead to significant maternal mortality. Usually, the presentation includes abdominal pain, fever and vaginal bleeding.

**Case presentation:**

We reported the case of a female in her twenties in her second trimester of pregnancy following unsafe abortion. The patient had abdominal pain, and laboratory investigations revealed anemia and leucocytosis. The patient opted for abortion as the foetus was identified as female by a service provider. Due to unsafe and illegal abortion, the patient developed complications of incomplete abortion and uterine rupture. She was successfully managed by emergency laparotomy followed by repair of uterine rupture and symptomatic management.

**Clinical discussion:**

Unsafe abortion can lead to complications such as incomplete abortion and uterine rupture. Complications due to abortion are more frequent if not performed by experienced surgeons. In our case, the manual vacuum and aspiration technique was used during the second trimester of pregnancy, which led to uterine perforation.

**Conclusion:**

Our case highlighted the importance of safe abortion practices and the approach to clinical management of complications of unsafe abortion. Also, global health problems such as unsafe abortion, illegal abortion, sex-selective abortion, and violation of ethical conduct need to be addressed to curb unsafe abortion.

## Highlights

• Unsafe abortion can lead to incomplete abortion and uterine rupture.
• MVA is recommended in the first trimester, and D&E is recommended in the second trimester.
• It is essential to counter the trend of sex-selective abortion, particularly in developing countries.

## 1 Introduction

The global estimate by WHO revealed that 73 million induced abortions happen each year, of which nearly 45% are unsafe, and 97% occur in developing countries [1]. Asia, particularly South and Central Asia, constitute more than 50% of unsafe abortions [1]. A study revealed that approximately 7.9% of maternal mortality was due to unsafe abortions [2]. A study in Africa revealed that death due to unsafe abortions accounted for about one-third of all maternal mortalities [3]. These patients usually present with fever, abdominal pain, and vaginal bleeding [4]. Furthermore, it can lead to complications such as incomplete abortion, uterine rupture, and traumatic injury to the genital tract [2,5]. Hence, timely intervention is needed to prevent mortality. Herein, we present the successful management of a female in her twenties Gravida 3 Parity 2 Living 1 Infant death 1 at 19 weeks of gestation presenting with abdominal pain following abortion.

## 2 Presentation of case

### 2.1 Presentation and history

A female in her twenties Gravida 3 Parity 2 Living 1 Infant death 1 at 19 weeks of gestation presenting to Gynae Emergency with a complaint of pain abdomen for 11 hours. The pain was present over the whole abdomen, severe and started following the manual vacuum and aspiration (MVA) procedure performed at a local clinic. On further ienquiry, she said she visited a local clinic for gender determination, where the foetus was identified as a female. As the patient and her family members did not want to have a female child, she opted for termination of pregnancy at the same clinic. After the procedure, she was given some intravenous analgesics for pain relief and referred to our hospital. On the route to our hospital, she passed urine and flatus. There was no history of nausea, vomiting, fever, excessive vaginal bleeding, or loss of consciousness. The patient has been married for 12 years. In her first pregnancy, she had to undergo an emergency lower segment caesarean section (LSCS) for meconium-stained liquor and gave birth to a healthy female baby. Currently, the child is of 5 years. In her second pregnancy, she gave birth to a female child of 2.5 kg weight through elective LSCS. The child died at 6 months of age due to some unknown cause. The patient was not using contraceptives, and the medical history did not reveal any chronic illness in the patient and family members. There was no history of any drug allergies, alcohol consumption, smoking or recreational drug use.

### 2.2 Physical examination

On general examination, she had pallor. Her vitals were: blood pressure of 100/80 mm of Hg, pulse rate of 102 beats per minute, respiratory rate of 24 cycles per minute, spo2 of 98%, and the temperature was 98.8° Fahrenheit. On abdominal examination, mild tenderness was present in the left hypogastric region, and bowel sounds were heard. Her size of the uterus corresponded to 22 weeks period of gestation. The rest of the systemic examinations were normal. On per speculum examination, a gestational sac was felt through the external os with pieces of essential fat present. On per vaginal examination, the uterus was 20–22 weeks in size, and a gestational sac-like structure was felt.

### 2.3 Laboratory findings

The initial laboratory investigations (Table 1) revealed anaemia and leucocytosis. Ultrasonography of the abdomen and pelvis revealed macerated foetus in the intrauterine cavity with no cardiac activity. Also, echogenic content was reported in the lower part of the endometrial cavity with posterior acoustic shadow; the posterior wall and right uterine wall were not delineated clearly. Ultrasonography could not rule out the possibility of uterine perforation.

**Table 1:** Laboratory investigations of the patient.

Investigations	Operative day	Post-operative Day 1
Complete Blood Count		
Haemoglobin (g/dl)	10.1	9.5
PCV (%)	32.5	30.1
Total leukocyte count (cells/mm^3^)	14700	152000
**Differential Leukocyte Count**		
Neutrophil (%)	86	90
Lymphocyte (%)	9	04
Monocyte (%)	5	04
Platelet Count (cells/mm^3^)	1,78,000	1,56,000
**Prothrombin Time** (second)	15	
**INR**	1.12	
**Urine RE/ME**		
Protein	Negative	
Sugar	Negative	
WBC (per HPF)	4–6	
R.B.C. (per HPF)	Not seen	
Epithelial Cells (per HPF)	2–3	
**Urine Culture and Sensitivity**	Sterile	
**Blood Grouping**	O positive	
**Random Blood Glucose** (mg/dl)	101	
**Serology**		
HBsAg	Negative	
HCV	Negative	
H.I.V.	Negative	
VDRL/RPR test	Non-reactive	
**Serum urea** (mg/dl)	22	16
**Serum Creatinine** (mg/dl)	0.9	0.6
**Liver Function Test**		
Total Protein (g/dl)	6.0	
Albumin (g/dl)	3.8	
Total Bilirubin (mg/dl)	0.5	
Conjugated Bilirubin (mg/dl)	0.1	
ALT (U/L)	12	
A.S.T. (U/L)	14	
A.L.P·(U/L)	63	
G.G.T. (U/L)	13	
**Serum Electrolytes**		
Sodium (mmol/L)	137	136
Potassium (mmol/L)	3.6	3.2

### 2.4 Assessment and intervention

**Fig. 1:**
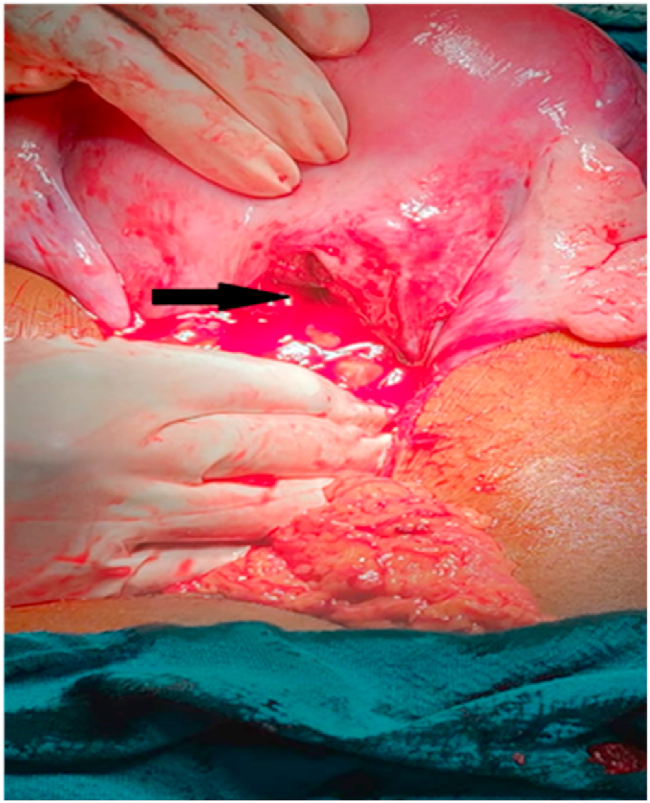
Uterine perforation (shown by arrow).

**Fig. 2:**
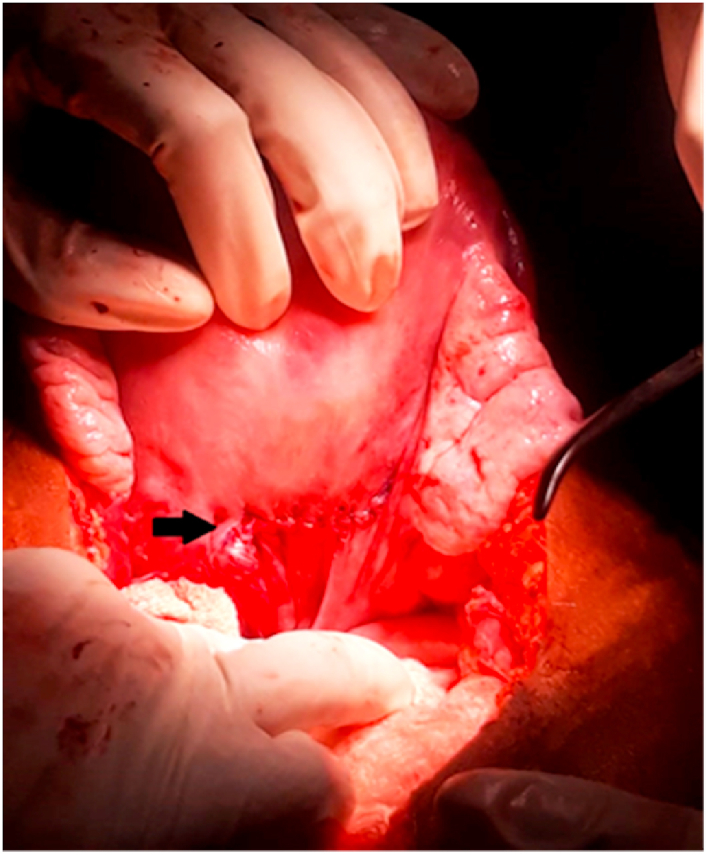
Repair of Uterine perforation (shown by arrow).

After reviewing the investigations, she was transferred to the operation theatre for emergency exploratory laparotomy. The procedure was performed at a tertiary care hospital by a senior consultant with more than ten years of experience. An epidural catheter and central venous pressure line were inserted. A midline vertical incision was given, and the abdominal cavity was opened in layers. Rent of 2 cm × 2 cm size was seen (Fig. 1) on the posterior wall of the uterus through which a bowel loop entered the uterine cavity. The bowel loop was released from the uterine cavity and examined for any perforation or tear by the surgery team. There was no perforation or tear in the bowel. A small tear was present in mesentery with no bleeding. Then the bowel loop was placed into the abdominal cavity. After that, the foetus was removed along with the placenta. The removed foetus was identified as male. Uterus was closed with vicryl suture (Fig. 2), followed by the closure of the rectus sheath with prolene suture. The skin was closed by applying stapler. An antiseptic bandage was applied, followed by vaginal toileting.

Tablet Misoprostol 800 μg was given per rectal. The patient was started on intravenous antibiotics Piperacillin plus Tazobactam 4.5 gm, Metronidazole 500mg, Ranitidine 50mg, Metoclopramide 10mg, Ketorolac 30mg, Paracetamol 1 gm were given three times a day. Also, Synthetic Oxytocin 20 Units in 3 pints of intravenous fluids was given. In addition, intravenous Ringer's Lactate 1 unit and 2 units of Normal Saline were given. She also received 1 pint of Whole Blood and 1 pint of Fresh Frozen Plasma. Then, the patient was shifted to the maternal intensive care unit, where she was kept under vigilant observation. She was kept nil per oral for 48 hours, and input and output charting was done.

### 2.5 Post-operative history

On her first post-operative day, she received intravenous potassium chloride 20 mEq in alternate 2 pints of intravenous fluids as she had hypokalaemia (Table 1). Intravenous Ketorolac was given when required, and she received 1 pint of packed cell blood. Intravenous Synthetic Oxytocin was stopped. The patient was encouraged to ambulate. On her second post-operative day, the urinary catheter was removed, and the drugs were continued. All her intravenous drugs were discontinued on the fifth post-operative day, and she was switched to oral Levocetirizine 5 mg and Paracetamol 1 gm. She was discharged on her sixth post-operative day.

### 2.6 Follow up

On follow-up after one month, the patient was in good health and doing well.

## 3 Discussion

Across the world, various sociological factors determine the sex preference of a child by the parents [[6], [7], [8]]. The countries in South-East Asia have primarily been patriarchal societies [6]. Hence, having a male child is preferred in these countries [[6], [7], [8], [9]]. Also, sex-selective abortion is rising globally, particularly in South-East Asia [1,[10], [11], [12], [13]]. This leads to a skewed birth rate and can be detrimental to the population control policy. Furthermore, in countries that prohibit sex-selective abortion, couples can seek illegal options and abort the foetus [9,13,14]. The couples may take the help of service providers lacking the required skills, leading to unsafe abortion practices. Illiteracy, all children being female, and social shame was also associated with unsafe abortions [15,16]. In our case, the foetus was misidentified as female and she already had two female children from her last two pregnancies, motivating the patient to undergo an illegal abortion [17,18]. Moreover, safe abortion practices were not followed [15] This also highlighted gross negligence and violation of legal and ethical boundaries, which needs to be addressed to limit such incidents in the future.

Unsafe abortion can lead to incomplete abortion, haemorrhage, uterine perforation and damage to the genital tract [2,5]. Studies in Pakistan revealed that the maternal mortality from unsafe abortion was nearly 34.9%, with uterine perforation, septicaemia and gastrointestinal injury being the common complications [3,14]. A study in Nigeria revealed that abdominal pain, fever and vaginal bleeding were the most common presenting symptoms in unsafe abortions [4]. A study revealed that the incidence of uterine perforation following unsafe abortion by Manual vacuum and aspiration was around 0.4% in a hospital setting in India [19]. Another study revealed that the chance of surgical abortion failure was more if done by health providers other than doctors [20]. In our case, the patient presented with abdominal pain and the abortion was performed by an unskilled service provider through manual vacuum aspiration during the second trimester of pregnancy. The manual vacuum and aspiration technique is the surgical choice for medical termination of pregnancy during the first trimester [21]. During the second trimester, the surgical approach of Dilatation and Evacuation is preferred over the medical approach [22]. However, in our case, the manual vacuum and aspiration technique was used during the second trimester of pregnancy, which led to uterine perforation.

This work has been reported in line with SCARE 2020 criteria [23].

## 4 Conclusion

Our case highlighted the approach to management of complications of unsafe abortion such as incomplete abortion and uterine rupture. Also, we identified four major global health problems: increasing trend of sex-selective abortion in south-east Asia, unsafe abortion leading to maternal complications, breach of ethical code of conduct, and sociological factors contributing to illegal abortion. It is necessary to address these factors to counter the global health problem of unsafe abortion, particularly in developing countries. Efforts are required to counter the gender-based power imbalance through women empowerment. Also, the government should act to expand safe abortion facilities and take measures to ensure that healthcare providers provide the optimum quality of service.

## Ethical approval

Ethical approval was not required for this case report.

## Sources of funding

The authors did not receive any funding for this manuscript.

## Author contributions

A.A. and A.G. drafted and critically revised the manuscript. P.Y. and P.R. critically revised the manuscript. All authors approved the final version of the manuscript and are accountable for all aspects of the work.

## Registration of research studies

1. Name of the registry: N/A.
2. Unique identifying number or registration ID: N/A.
3. Hyperlink to your specific registration (must be publicly accessible and will be checked): N/A.

## Guarantor

Punita Yadav is the Guarantor.

## Provenance and peer review

Not commissioned, externally peer-reviewed.

## Consent of patient

Written informed consent was obtained from the patient for publication of this case report and accompanying images. A copy of the written consent is available for review by the Editor-in-Chief of this journal on request.

## Declaration of competing interest

The authors have no conflict of interests to declare.
